# Higher cut-off serum procalcitonin level for sepsis diagnosis in metastatic solid tumor patients

**DOI:** 10.1186/s13104-018-3204-1

**Published:** 2018-01-30

**Authors:** Segal Abdul Aziz, Erni Juwita Nelwan, Lugyanti Sukrisman, Suhendro Suhendro

**Affiliations:** 1 Department of Internal Medicine, Medical Faculty University of Indonesia-Cipto Mangunkusumo General Hospital, Jalan Diponegoro No. 71, Jakarta, 10430 Indonesia; 2 Division of Tropical Medicine and Infectious Disease, Department of Internal Medicine, Medical Faculty University of Indonesia-Cipto Mangunkusumo General Hospital, Jalan Diponegoro No. 71, Jakarta, 10430 Indonesia; 3 Division of Hematology and Medical Oncology, Department of Internal Medicine, Medical Faculty University of Indonesia-Cipto Mangunkusumo General Hospital, Jalan Diponegoro No. 71, Jakarta, 10430 Indonesia

**Keywords:** Procalcitonin, Metastasis, Sepsis, Solid tumor

## Abstract

**Objective:**

The current study aimed to know procalcitonin levels in patients with metastatic tumor, and to discover the cut-off point for sepsis in this population. A cross-sectional study was conducted with patients with solid tumor. Sepsis and systemic inflammation response syndrome (SIRS) were identified using clinical, laboratory, and microbiological criteria. The cut-off point was determined using receiver operating characteristic (ROC) curve.

**Results:**

A total of 112 subjects enrolled in this study, 51% male, mean age 47.9 ± 12.47 years. Among 71 (63.4%) patients who had metastasis, 36 (32.1%) had sepsis and 6 (5.3%) experienced SIRS. In the absence of sepsis, the procalcitonin levels were significantly higher in patients with metastatic tumor compared to those without [0.25 ng/mL (0.07–1.76) vs. 0.09 ng/mL (0.03–0.54); p < 0.001]. The ROC curve showed that levels of procalcitonin for sepsis in metastatic solid tumors were in the area under curve (AUC) [0.956; CI 0.916–0.996]. Cut-off point of procalcitonin for sepsis was 1.14 ng/mL, Sn 86%, and Sp 88%. Thus, the results show that metastatic tumor affects the patients’ procalcitonin level, even in the absence of sepsis. The cut-off point of procalcitonin level for diagnosing sepsis in the meta-static solid tumor was higher compared to the standard value.

**Electronic supplementary material:**

The online version of this article (10.1186/s13104-018-3204-1) contains supplementary material, which is available to authorized users.

## Introduction

Infection is one of the most important complications in patients with cancer, frequently leading to high rates of morbidity and mortality. The risk of infection is 10 times higher in cancer patients compared to those without malignancy. Decreasing of both specific and non-specific immune response is believed to be the cause of the vulnerability [1–3]. Sepsis, a systemic inflammation response to infection, frequently ends in a more catastrophic situation especially in patients with malignancy, if it is not diagnosed and treated immediately. Some studies showed that sepsis causes 50% of mortality in malignancies, 40–60% of which were due to septic shock, and it does not depend on the type of malignancies [4, 5].

Unfortunately, it is not always easy to diagnose sepsis in cancer patients. Fever and leukocytosis are common in patients with tumor even in the absence of infection. While infiltrate in chest radiography can be a sign of lung metastasis, definitive microbiological investigation usually takes a long time to get the result. As a result, specific biomarkers are critical for diagnosing sepsis in this population [6, 7]. Procalcitonin (PCT), a pre-hormone of calcitonin, has been established as specific marker of sepsis, but its level can be influenced by some conditions, such as in solid tumor [8]. Metastasis tumor, through complex processes involving interleukin (IL)-6, IL-2, and tumor necroting factors (TNF), is hypothesized as a component that involves increasing PCT levels, and found to be not dependent on the type of tumor, except in medullary thyroid carcinoma and neuroendocrine lung tumor [9, 10]. However, one study from Giovanella et al. [11] showed that there was no increase in PCT levels even in stage IV patients with metastatic tumor.

This study aimed to find out whether metastasis tumor increases PCT levels in non-septic patients and determine its diagnostic value for sepsis in patients with metastatic tumor.

## Main text

### Methods

#### Study design

This cross-sectional study was conducted in Cipto Mangunkusumo National General Hospital, Jakarta, Indonesia, from August to November 2015. The subjects were chosen consecutively from the surgical and non-surgical wards, emergency department, and outpatients.

#### Study participants

Study participants were adult patients with any solid tumor who had a complete staging, confirmed by histological and imaging examination. Before becoming enrolled in this study, informed consent forms were completed by each subject. All subjects underwent history taking and physical examination, as well as chest X-ray, and laboratory examination [complete blood count, blood urea nitrogen (BUN), creatinine, alanine aminotransferase (ALT), aspartate aminotransferase (AST), blood glucose, urine analysis, PCT, and C-reactive protein (CRP)]. Tests of liver ultrasound and hepatitis markers were done in patients with increased ALT, AST or any suspicion of cirrhosis. Patients with medullary thyroid carcinoma, neuroendocrine lung cancers, any previous antibiotics therapy within last 72 h, shock, and/or any condition that can influence serum PCT level (recent surgical, multiple trauma, resuscitation, dialysis, cirrhosis, or received any colony stimulating factors) were excluded. We checked blood and site-specific culture for all subjects who met SIRS diagnosis, according to The American College of Chest Physician/Society of Critical Care Medicine (ACCP/SCCM) 2001 sepsis criteria [two or more of the following: body temperature > 38.5 or < 35.0 °C; heart rate of > 90 beats per minute; respiratory rate of > 20 breaths per minute or PaCO_2_ (arterial partial pressure of CO_2_) of < 32 mmHg; and white blood cell count of > 12,000, < 4000 cells/mL, or > 10% immature (band) forms] [12]. Sepsis was indicated if SIRS plus infection were proved clinically or through positive culture.

#### Laboratory examination

Blood was drawn by a trained nurse and processed according to hospital standard protocol. PCT levels were measured using BRAHMS PCT KRYPTOR^®^ tool, that was calibrated as specified by the manufacturer’s protocol. This tool has a lower limit of quantification of 0.02 ng/mL. Standard media transport was used for site-specific culture (sputum, urine, feces, etc.). We used BACTEC bottles for blood culture media. The laboratory examinations were conducted by the laboratory staff who were not included in the investigator team. Moreover, all examinations were run through the appropriate machines which were calibrated and the results generated automatically. Thus, we considered that the investigators were blinded.

#### Tumor staging

Tumor staging was done according to the American Joint Committee on Cancer (AJCC) criteria for each kind of solid tumor and reviewed by a certified oncologist. All tumors were already confirmed histopathologically and examined by an experienced pathologist from the Department of Pathology Cipto Mangunkusumo Hospital. In this study, we grouped type of tumor into head and neck, colorectal, musculoskeletal, breast, lung, genitourinary, gynecology, pancreatobiliary, and thyroid. Metastatic tumor was defined as distant metastasis lesion discovered by imaging examination [magnetic resonance imaging (MRI), computed tomography (CT) scan, X-ray, and/or ultrasound] or cytology. Locally advanced or locally invasive tumor was not considered as metastasis.

#### Statistical analysis

All data were analyzed using statistical package for the social science (SPSS) program version 20. The results were reported as median (minimum–maximum) and differences in leukocyte, PCT, and CRP were presented using the Mann–Whitney test. The *p* value < 0.05 was considered as statistically significant. The cut-off point of PCT level for sepsis in subjects with metastatic tumor was done by AUC analysis from ROC curve.

### Results

There were 112 subjects, mean of age 48 years old, enrolled in the study of a total 128 patients with solid tumors who came to the hospital. The difference of male and female proportion was subtle (50.9% vs. 49.1%), while the mean age for males was 50 ± 13.7 years old and females was 45.8 ± 10.8 years old. Subjects were divided into metastatic and non-metastatic groups. We identified sepsis and non-sepsis subjects from each group as seen in Additional file 1: Figure S1. Subjects with SIRS without any proven infection were classified into the non-sepsis subgroup.

Head and neck (e.g. nasopharynx, hypopharynx, oropharynx) were the most common type of tumor found, followed by colorectal, gynecological (e.g. ovarian, cervix), breast, lung, and pancreatobiliary. There were 87 subjects (78%) included in stage IV according to AJCC criteria, and 71 (63%) of them were confirmed to have distant metastasis. A total of 56 subjects met SIRS criteria, and 45 of them were diagnosed sepsis, mostly caused by pneumonia (53%), while bacteremia was only found in six subjects (13%). Subjects’ characteristics are provided in Table 1.

**Table 1:** Characteristics of subjects of study

Characteristics	n = 112
Age, years, mean (SD)	47.9 (12.47)
Male, n (%)	57 (50.9)
Tumor group, n (%)	
Head and neck	26 (23.2)
Colorectal	20 (17.9)
Musculoskeletal	8 (7.1)
Breast	12 (10.7)
Lung	12 (10.7)
Genitourinary	8 (7.1)
Gynecology	14 (12.5)
Pancreatobiliary	10 (8.9)
Thyroid	2 (1.8)
Stage (according to AJCC), n (%)	
I	3 (2.7)
II	5 (4.5)
III	17 (15.2)
IV	87 (77.7)
Metastasis, n (%)	71(63.4)
SIRS, n (%)	56 (50)
Sepsis, n (%)	45 (40.2)
Severe sepsis, n (%)	10 (8.9)
Infection site, n (%)	
Pneumonia	24 (21.4)
UTI	11 (9.8)
Intra-abdominal	4 (3.5)
Skin and soft tissue	8 (7.1)
Blood	6 (5.3)
Leukocyte, /μL, median (min–max)	12,100 (1100–67,600)
CRP, mg/L, median (min–max)	23.5 (0.5–271.4)
PCT, ng/mL, median (min–max)	0.64 (0.03–921.4)

*AJCC* American Joint Committee on Cancer, *UTI* urinary tract infection, *CRP* C-reactive protein, *PCT* procalcitonin

In the non-sepsis sub-group, PCT levels were significantly higher in metastasis subjects compared to those without metastasis [0.25 ng/mL (0.07–1.76) vs. 0.09 ng/mL (0.03–0.54); *p* < 0.05], while levels of CRP and leukocyte were not significantly different. Meanwhile, in the sepsis sub-group, there was no significant difference in either PCT, CRP, or leukocyte between metastasis and non-metastasis subjects (Table 2).

**Table 2:** Serum PCT, CRP, and leukocyte in all study groups

Sepsis biomarker	Metastasis		Non-metastasis	
	Sepsis	Non-sepsis	Sepsis	Non-sepsis
	n = 36	n = 35	n = 9	n = 32
Leukocyte, /μL, median (min–max)	19,500 (4160–67,600)**	8860 (2930–24,910)	13,830 (2050–48,340)	7730 (1100–22,680)
CRP, mg/L, median (min–max)	112.5 (16.2–259.3)**	13.6 (0.8–210.5)	143.1 (51–271.4)	5.8 (0.5–209.8)
PCT, ng/mL, median (min–max)	3.48 (0.66–189.4)**	0.25 (0.07–1.76)*	2.92 (1.1–921.4)	0.09 (0.03–0.54)

Mann–Whitney test; *p < 0.05 vs. group non-metastasis non-sepsis; **p < 0.05 vs. group metastasis non-sepsis

**Fig. 1:**
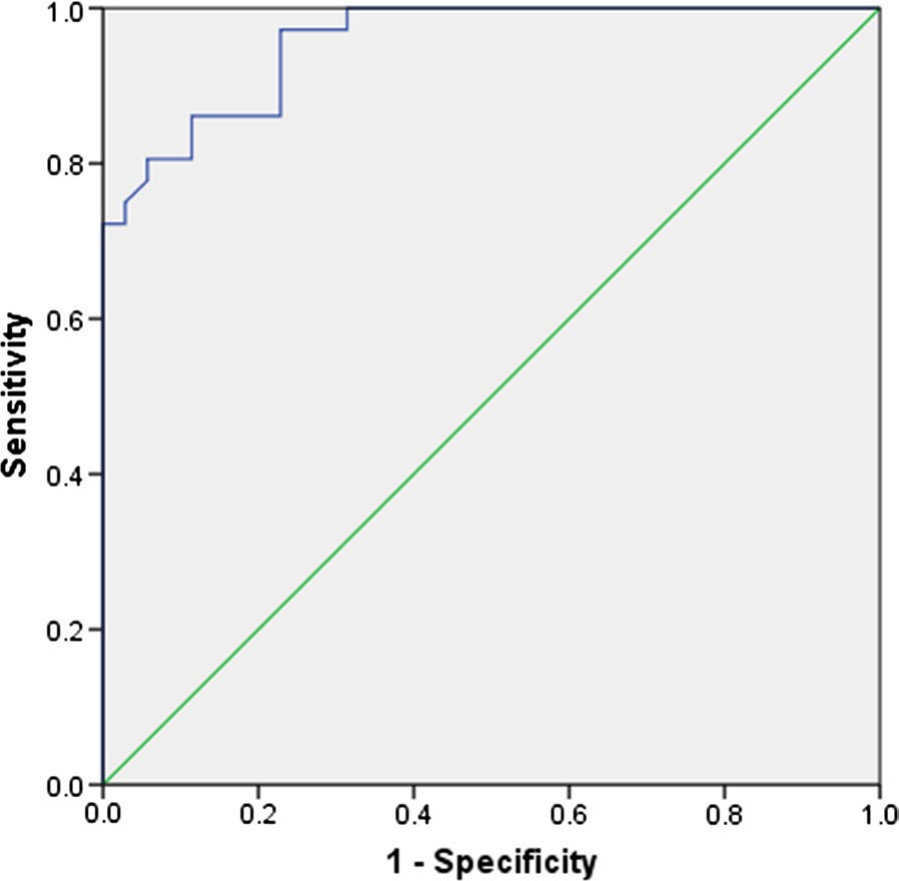
Procalcitonin ROC curve for sepsis in metastatic solid tumor

Using the ROC curve of PCT level (Fig. 1), we found the cut-off for diagnosing sepsis was 1.14 ng/mL and the AUC 0.956 (CI 0.916–0.996), as shown in Additional file 2: Table S1.

### Discussion

As demonstrated in our study, patients with advanced stage tumor have the highest proportion among all tumor cases, associated with the lack of early detection or screening program. This issue has not only been described for developing countries, but also some developed countries [13–16]. Patients usually come to the hospital due to their complications, such as sepsis, bleeding, or dehydration, instead of the tumor itself [16, 17]. As shown in this study, about 40% of the subjects were diagnosed sepsis. We found that the head and neck cancer proportion was the highest of all types of solid tumor. This pattern may be associated with demographics, since head and neck tumor is the fourth most common solid tumor in Indonesia [13].

A number of studies have shown that leukocytosis can occur in patients with solid tumor even without infection, thus making it a non-reliable biomarker for infection in this group. The increase usually occurs in certain type of tumors, such as gastric, lung, pancreas, brain, cervix, and malignant melanoma, or due to paraneoplastic syndrome [7, 18, 19]. Our subjects were dominated by head and neck tumor and colorectal cancer, thus leukocytosis rarely happened without infection, and that fact might help to explain why the difference in leukocyte count was statistically significant between sepsis and non-sepsis.

In the absence of sepsis, PCT levels of patients with metastatic tumor were significantly higher than those without metastasis, in contrast with CRP or leukocyte, where the increases in those markers were not statistically significant [13.6 mg/L (0.8–210.5) vs. 5.8 mg/L (0.5–209.8), *p* = 0.370, and 8860/μL (2930–24,910) vs. 7730/μL (1100–22,680), *p* = 0.629], respectively. However, our purpose was not to demonstrate PCT as a marker of metastasis, but this result indicates that metastasis is an important factor that can increase PCT levels in patients with solid tumor. Various studies have already proved that PCT levels are increased in patients with solid tumor, but they failed to show significant increases in metastatic subject compared to those without metastasis [20, 21].

This study has not been able to determine whether the increase of PCT levels is related to the site of metastasis, since PCT is mainly produced by liver. However, PCT levels were also increased in malignant pleural effusion range from 0.1 to 0.34 ng/mL, and with that result we believe the increased level of PCT does not depend on the site of metastasis [22–24].

This study also intends to determine the diagnostic value of PCT in the patients with metastatic tumor as a sepsis marker, and whether the standard cut-off level for sepsis (0.5 ng/mL) is still reliable in this population. From the AUC analysis, PCT showed a good performance [AUC 0.956, (CI 0.916–0.996)]. We also found that the optimum cut-off is 1.14 ng/mL with sensitivity 86% and specificity 88%. Some previous studies showed that in the malignancy population, the optimal cut-off level was still 0.5 ng/mL with moderate performance (Sn 21–92.9% and Sp 45–92%) [15, 16].

Clinical applicability of this new cut-off is clear, for example, in a metastatic breast cancer patient who comes to the ER with dyspnea, tachycardia, leukocytosis, chest X-ray shows unilateral effusion, and PCT level 0.75 ng/mL. We are fairly certain that the SIRS in this patient is not caused by infection (sepsis) and she does not need aggressive antibiotics therapy. However, PCT is only a biomarker and it is not a gold standard for diagnosing sepsis, which means clinical judgment still plays the primary role to decide the best treatment for the patient. Furthermore, the development of new sepsis guidelines and its definition, also influence our paradigm in deciding the diagnosis of sepsis [25].

## Limitations

Most of the study participants were from the medical ward, and this limitation can cause some biases. This source of medical record was also the cause why the demographic data of the type of tumor is different from the national or regional characteristics. We were also still using sepsis criteria from ACCP/SCCM 2001, which might influence the result of cut-off point.

Additionally, we had insufficient number of non-metastasis subjects in the sepsis sub-group (n = 9), and this limitation might help to explain why the sepsis biomarkers were not significantly different compared to the metastasis subjects.

## Additional files

**Additional file 1: Figure S1.** Study algorithm; description: describing how many subject enrolled, how many were excluded, and how was the divisions of the subject group.

**Additional file 2: Table S1.** Diagnostic value of PCT for diagnosing sepsis in metastatic tumor patient; description: describing the performance of PCT as a sepsis biomarker in metastatic tumor patient.

## Abbreviations

ACCP/SCCM: The American College of Chest Physician/Society of Critical Care Medicine
AJCC: American Joint Committee on Cancer
ALT: alanine aminotransferase
AST: aspartate aminotransferase
AUC: area under curve
BUN: blood urea nitrogen
CRP: C-reactive protein
CT: computed tomography
IL: interleukin
MRI: magnetic resonance imaging
PCT: procalcitonin
ROC: receiver operating characteristic
SIRS: systemic inflammation response syndrome
SPSS: statistical package for the social science
TNF: tumor necroting factor
