# Supplementary figures and images for: Higher cut-off serum procalcitonin level for sepsis diagnosis in metastatic solid tumor patients
Source: BMC Res Notes. 2018 Jan 30;11:84. doi: 10.1186/s13104-018-3204-1 (PMC5791197; doi:10.1186/s13104-018-3204-1)

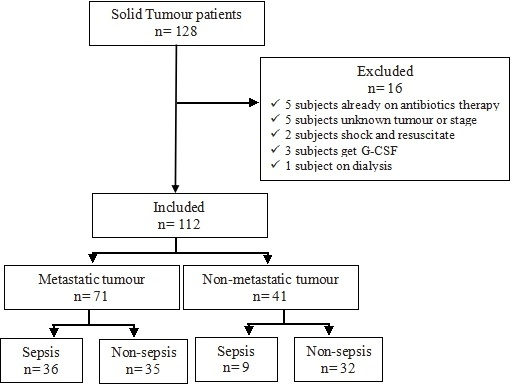

Supplement: Supplementary file 1 — Additional file 1: Figure S1. Study algorithm; description: describing how many subject enrolled, how many were excluded, and how was the divisions of the subject group. [file 13104_2018_3204_MOESM1_ESM.docx]
