# Supplementary material for: Higher cut-off serum procalcitonin level for sepsis diagnosis in metastatic solid tumor patients
Source: BMC Res Notes. 2018 Jan 30;11:84. doi: 10.1186/s13104-018-3204-1 (PMC5791197; doi:10.1186/s13104-018-3204-1)
Supplement: Supplementary file 2 — Additional file 2: Table S1. Diagnostic value of PCT for diagnosing sepsis in metastatic tumor patient; description: describing the performance of PCT as a sepsis biomarker in metastatic tumor patient. [file 13104_2018_3204_MOESM2_ESM.docx]

**Table S1. Diagnostic value of PCT for diagnosing sepsis in metastatic tumor patient**

| **PCT**  **ng/mL** | **AUC** | **Sensitivity** | **Specificity** | **NPV** | **PPV** | **PLR** | **NLR** |
| --- | --- | --- | --- | --- | --- | --- | --- |
| 1.14 | 0.956 | 0.86 | 0.88 | 0.86 | 0.88 | 7.53 | 0.15 |

PCT: procalcitonin; AUC: area under curve; NPV: negative predictive value; PPV: positive predictive value; PLR: positive likelihood ratio; NLR: negative likelihood ratio
